# Anatomical Variations of the Temporalis Muscle: A Magnetic Resonance Imaging–Based Study

**DOI:** 10.1155/bmri/3179553

**Published:** 2026-01-30

**Authors:** Adrian Okoń, Ingrid C. Landfald, Michał Podgórski, Roman Frach, George Triantafyllou, Łukasz Olewnik

**Affiliations:** ^1^ Department of Clinical Anatomy, Mazovian Academy in Płock, Płock, Poland; ^2^ Department of Radiology, Medical University of Lodz, Płock, Poland, umed.pl; ^3^ Department of Anatomy, School of Medicine, Faculty of Health Sciences, National and Kapodistrian University of Athens, Athens, Greece, uoa.gr

## Abstract

**Introduction:**

The temporalis muscle is a key masticatory muscle involved in jaw movement, specifically in the elevation and retraction of the mandible. This muscle has a complex anatomical structure, which includes both superficial and deep layers that are vital for its function. Variability in the muscle’s morphology can influence its efficiency in chewing and may play a role in several clinical conditions, such as temporomandibular joint disorders (TMDs) and bruxism. Understanding the anatomical variations in the temporalis muscle is essential for improving diagnostic accuracy and treatment approaches.

**Material and Methods:**

This study included 34 female participants (age: median 48.5 [36–64] years) and 24 male participants (age: median 47.5 [37–68] years). MRI scans were used to assess the anatomy of the temporalis muscle, focusing on the presence of both superficial and deep muscle layers. The presence of a longitudinal muscle core was also examined to identify anatomical variations. Cases were classified based on the presence of both layers and the extent of hypoplasia in the posterior portion of the superficial layer. Descriptive statistics are reported as median and the first and third quartiles (Q1–Q3), and group differences were assessed with nonparametric tests.

**Results:**

The study revealed that the temporalis muscle exhibited significant anatomical variability across participants. In six cases, only the deep layer was present, with symmetry in one female and one male. In 26 cases, the posterior portion of the superficial layer was hypoplastic, appearing as a thin muscular layer. Hypoplasia was significantly more common in males (*p* = 0.02168). Based on these observations, the muscle was classified into three groups: (1) muscles with both layers intact (72.4%), (2) muscles with both layers but with a hypoplastic posterior portion of the superficial layer (22.4%), and (3) muscles with only the deep layer (5.2%).

**Conclusion:**

This study provides new insights into the anatomical variability of the temporalis muscle, highlighting differences in the superficial and deep layers and the prevalence of hypoplasia in the posterior portion of the superficial layer. These findings have important clinical implications, as they can improve radiological diagnostics and aid in the accurate identification of muscle variations. Understanding these variations enhances the ability to diagnose and treat temporomandibular disorders and related conditions more effectively, offering more personalized care for patients.

## 1. Introduction

The temporalis muscle (TM) is one of the major muscles of mastication, located on the side of the head, covering the temporal bone. It originates from the temporal fossa and the temporal fascia and inserts into the coronoid process of the mandible. The TM consists of a superficial and a deep portion. This layered structure reflects differences in fiber orientation and function, with both parts contributing to mandibular elevation and retraction. These structural characteristics allow the TM to efficiently assist in the chewing process [1].

Functionally, the TM plays a critical role in elevating the mandible and closing the jaw during mastication. The anterior fibers are primarily responsible for rapid elevation of the mandible, while the posterior fibers assist in retraction. Together with the masseter and pterygoid muscles, the TM contributes to effective grinding, crushing, and chewing. Additionally, the TM stabilizes the temporomandibular joint (TMJ), providing support during functional movements of the jaw, such as speaking and swallowing [2, 3].

Anatomical variations of the TM are commonly observed and may have important functional implications. Differences in the shape, size, and fiber orientation of the muscle have been noted, including accessory muscle slips or altered fiber arrangement [4]. Hypertrophy of the TM can occur in response to conditions such as bruxism, while atrophy or weakness of the muscle may be associated with jaw instability or masticatory dysfunction [1, 5].

Radiological diagnostics, particularly through imaging techniques such as magnetic resonance imaging (MRI) and computed tomography (CT), play a crucial role in assessing the TM. MRI offers high‐resolution imaging of soft tissue structures, allowing for detailed visualization of size, shape, and any pathological changes such as hypertrophy or atrophy [1]. CT imaging provides additional information regarding surrounding bony structures. Both imaging techniques are invaluable for diagnosing conditions affecting the TM, such as temporomandibular joint disorders (TMDs), myofascial pain syndrome, or traumatic injuries [6].

The clinical relevance of the TM is significant due to its vital role in mastication and its involvement in several pathological conditions. Dysfunction, hypertrophy, or atrophy of the muscle may lead to symptoms such as orofacial pain, compromised mastication, or cosmetic concerns [3, 5]. Accurate radiological assessment is therefore essential to guide diagnosis and treatment planning. By combining anatomical insight with imaging modalities, clinicians can ensure targeted and individualized patient care [7, 8].

This publication is aimed at providing a comprehensive analysis of the morphological variability of the TM using MRI. Through this investigation, we seek to examine the anatomical differences and their potential implications for clinical practice, particularly in the diagnosis and treatment of TMD. By analyzing MRI data, we aim to offer new perspectives on the muscle’s function and how its variations may contribute to both normal and pathological conditions.

## 2. Material and Methods

### 2.1. Study Design and Ethics

This retrospective cross‐sectional study analyzed routine brain MRI examinations. The cohort comprised 58 examinations, yielding 116 hemifaces (both sides included per subject). Adults of both sexes were referred mainly for vertigo or headache, and none had pathology involving the masticatory/facial region. The study complied with the Declaration of Helsinki and was approved by the Bioethics Committee at the Medical University of Lodz (Approval No. RNN/39/25/KE).

### 2.2. Eligibility Criteria

Inclusion required complete coverage of the masticatory apparatus and diagnostic image quality. Exclusion criteria were prior maxillofacial surgery or trauma, inflammatory or neoplastic processes involving the region of interest, and pronounced artefacts (motion‐related or caused by dental fillings).

### 2.3. MRI Acquisition

All scans were acquired on a Philips Ingenia 3.0T, with a Philips dStream 32‐channel head coil.

• T1‐weighted 3D (for morphometry): isotropic voxel 1.0 mm; FOV: 220; TR: 7.0 ms; TE: 3.2 ms; TI: 643 ms; FLIP ANGLE: 8 deg; ACQ Matrix *M* × *P*: 220^∗^220; acquisition time: 2:10; acquisition plane: SAG; acceleration: AI CS‐ SENSE; TFE factor: 204.
• T2‐weighted (reference): FOV: 230; TR: 4000 ms; TE: 128 ms; FLIP ANGLE: 90 deg; ACQ MATRIX *M*∗*P*: 356^∗^356; acquisition time: 2:24; acquisition plane: AXIAL acceleration: CS‐SENSE; TFE factor: 35; Slice thickness: 4 mm. T2 served as an anatomical reference for cortical bone and tendons/tendinous septa and was used for measurements whenever T1‐3D delineation was insufficient (e.g., suboptimal muscle contrast or too small field of view).

### 2.4. Classification Approach

Classification was specified a priori into three types (see Results section for full definitions). Layer identification was performed on standardized multiplanar reconstructions (MPRs) using the longitudinal muscle core as the landmark. Posterior superficial hypoplasia was defined a priori as a superficial layer no thicker than the longitudinal muscle core at the corresponding plane. Two experienced raters, blinded to sex and side, classified all sides independently, and resolved disagreements by consensus. Cases lacking a superficial layer were handled as described below.

### 2.5. Morphometric Variables

Measured variables included the following: (1) thickness at the level of the middle cranial fossa (MCF) for superficial and deep layers (where present); (2) maximum thickness of each layer; (3) length at the MCF level (overall muscle length)†; (4) maximum length and maximum height of the deep layer; (5) length of the coronoid process; and (6) distance from the coronoid apex to the temporalis attachment.

† “Length at the MCF level” denotes overall muscle length measured in that plane (not layer‐specific).

### 2.6. Measurement Protocol and Quality Control

MPRs were standardized to AC–PC line to ensure consistent slicing. Measurements were performed in RadiAnt DICOM Viewer; Version 2024.1 (64‐bit) by two independent raters using harmonized window/zoom presets; discrepant markings were resolved by consensus prior to analysis. Image sets not meeting quality criteria were excluded before measurements.

### 2.7. Handling of Absent Layers

For configurations without a superficial layer, parameters pertaining to that layer were recorded as NA (layer absent). Overall metrics applicable to all configurations (e.g., length at the MCF plane) were analyzed across configurations.

### 2.8. Statistical Analysis

All analyses were performed in Statistica 13 (TIBCO Software Inc., 2017). Data distributions were evaluated with the Shapiro–Wilk test. Because several variables departed from normality, between‐group comparisons used nonparametric tests: Mann–Whitney *U* (female vs. male), Wilcoxon signed‐rank (right vs. left), and Kruskal–Wallis (across temporalis types). Associations were examined with Spearman’s rank correlation (*ρ*). Descriptive results in Tables 1 and **2** are presented as median and the first and third quartiles (Q1–Q3). Two‐tailed *p* values are reported with *α* = 0.05.

**Table 1:** Morphological data across temporalis types.

**Parameter**	**Type 1**	**Type 2**	**Type 3**	**p**
Superficial (absent in Type 3)				
Thickness (level of middle cranial fossa)	4.7 (3.8–5.4)	5.4 (3.8–6.7)	—	0.1209
Length (level of middle cranial fossa)†	91.5 (88.4–95.8)	92.1 (84.9–96.9)	88.0 (81.3–95.5)	0.4648
Thickness (maximum)	6.45 (5.6–7.3)	6.75 (5.8–7.3)	—	0.5053
Deep				
Length (maximum)	101.8 (97.1–109.0)	105.3 (98.7–112.1)	100.2 (98.6–102.1)	0.2861
Height (maximum)	89.25 (83.45–95.6)	87.8 (81.6–93.8)	89.0 (82.3–92.7)	0.6864
Thickness (level of middle cranial fossa)	6.8 (6.05–7.75)	7.75 (6.4–8.9)	8.6 (8.2–9.0)	0.0048
Thickness (maximum)	8.25 (7.4–8.9)	9.2 (7.6–10.0)	9.85 (9.3–10.4)	0.0021
Coronoid process				
Length of the coronoid process	20.55 (19.0–22.1)	21.2 (19.3–23.5)	21.3 (19.9–24.7)	0.4121
Distance of the attachment from the top of the coronoid process	9.5 (8.1–12.1)	10.65 (7.9–13.3)	10.25 (7.8–13.9)	0.6518

*Note:* Values are median (Q1–Q3). *p* from Kruskal–Wallis. (— indicates a layer absent in Type 3; † overall length measured at the MCF plane, not layer‐specific).

**Table 2:** Sex‐ and side‐specific morphometric data.

**Parameter**	**Female**	**Male**	**p** **(F vs. M)**	**Right**	**Left**	**p** **(R vs. L)**
Superficial						
Thickness (level of middle cranial fossa)	4.45 (3.25–5.3)	4.95 (3.9–6.6)	0.0294	4.95 (3.3–5.9)	4.9 (3.5–5.4)	0.5254
Length (level of middle cranial fossa)	90.35 (86.4–92.55)	95.5 (90.85–100.6)	< 0.0001	92.2 (88.2–96.3)	90.95 (87.0–95.3)	0.3637
Thickness (maximum)	5.95 (5.1–6.75)	6.7 (6.05–7.80)	0.0012	6.45 (5.5–7.4)	6.3 (5.6–7.0)	0.4901
Deep						
Length (maximum)	99.55 (94.1–103.55)	108.0 (101.85–117.5)	< 0.0001	102.45 (98.6–110.2)	101.05 (95.8–107.2)	0.2816
Height (maximum)	87.7 (81.5–91.45)	92.0 (86.45–99.3)	0.0005	89.75 (83.0–95.8)	88.45 (82.3–93.3)	0.3522
Thickness (level of middle cranial fossa)	6.95 (6.0–8.1)	7.35 (6.35–8.45)	0.1670	7.1 (5.9–8.4)	7.2 (6.2–8.1)	0.8619
Thickness (maximum)	8.4 (7.55–9.1)	8.4 (7.55–10.05)	0.2881	8.45 (7.8–9.6)	8.4 (7.4–9.5)	0.6076
Coronoid process						
Length of the coronoid process	19.9 (18.05–21.65)	21.7 (20.3–23.7)	0.0003	20.65 (19.5–22.7)	20.75 (18.4–22.2)	0.3044
Distance of the attachment from the top of the coronoid process	9.45 (7.7–11.95)	10.6 (8.15–13.05)	0.1749	10.1 (8.2–12.3)	9.4 (7.6–12.6)	0.2141

*Note:* Values are median (Q1–Q3). *p* from Mann–Whitney *U* (female vs. male) and Wilcoxon signed‐rank (right vs. left); two‐tailed, *α* = 0.05.

## 3. Results

The study included 34 female participants (age: median 48.5 [36–64] years) and 24 male participants (age: median 47.5 [37–68] years).

On MPRs, superficial and deep layers were identified using the longitudinal muscle core as a landmark. A deep‐only configuration (absence of the superficial layer) was observed in six muscles across four subjects (two female and two male): It was bilateral in two subjects (one female and one male) and unilateral in the remaining 2.

Among muscles with both layers present, the posterior portion of the superficial layer was hypoplastic in 26 muscles, appearing as a thin muscular lamina on the aponeurosis. Hypoplasia was defined as a superficial layer no thicker than the longitudinal muscle core at the corresponding plane. This involved six female subjects (four bilateral) and nine male subjects (seven bilateral) and was more common in males (*p* = 0.02168).

Based on these observations, the temporalis was classified into three types used throughout the analyses:

• **Type 1**—both layers intact (84 muscles; 72.4%)
• **Type 2**—both layers present with posterior superficial hypoplasia (26 muscles; 22.4%)
• **Type 3**—deep‐only configuration (6 muscles; 5.2%)

**Figure 1:**
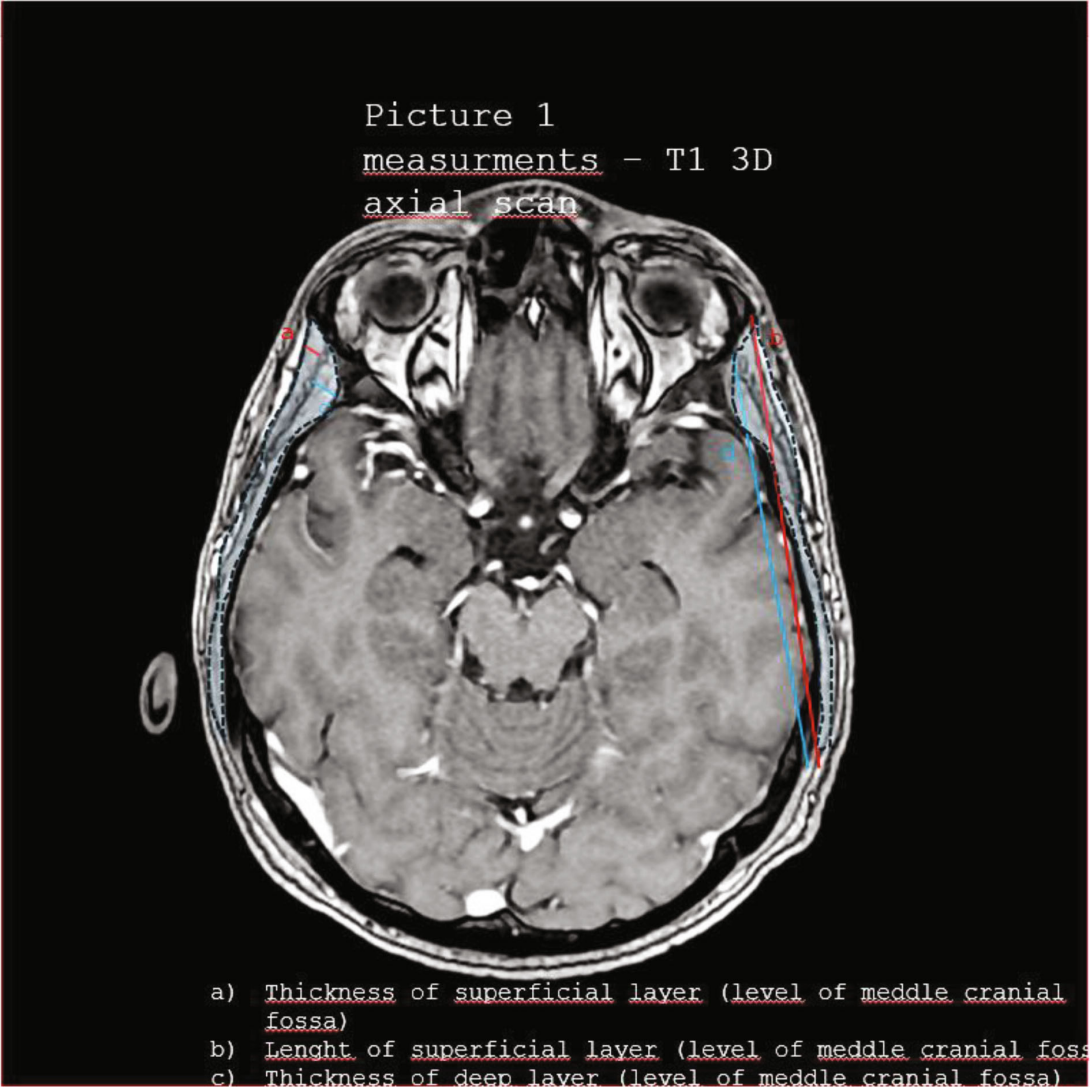
Measurements—T1 3D axial scan. (a) Thickness of superficial layer (level of meddle cranial fossa). (b) Length of superficial layer (level of meddle cranial fossa). (c) Thickness of deep layer (level of meddle cranial fossa). (d) Length of deep layer (level of meddle cranial fossa).

**Figure 2:**
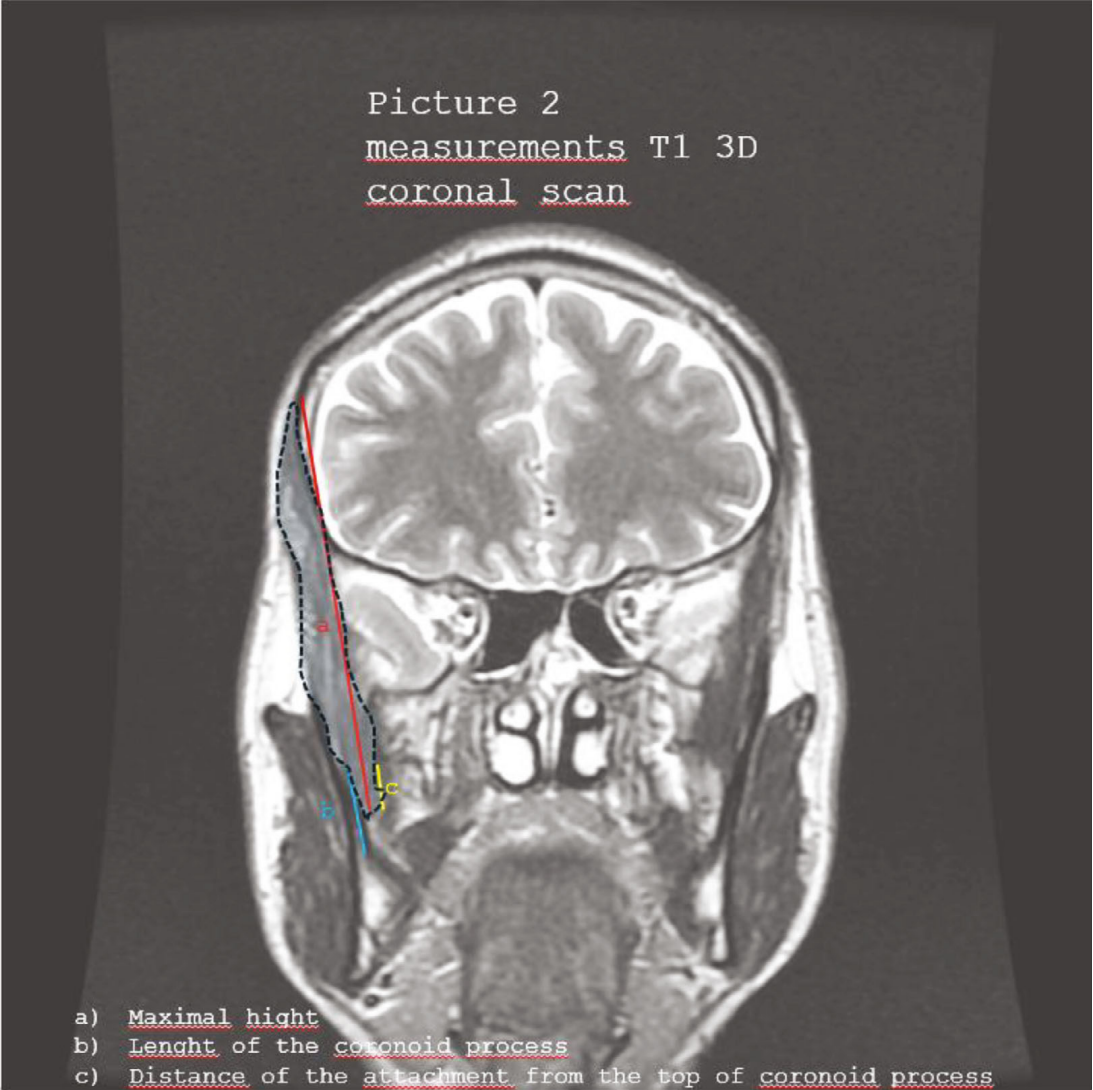
Measurements T1 3D coronal scan. (a) Maximal height. (b) Length of the coronoid process. (c) Distance of the attachment from the top of coronoid process.

**Figure 3:**
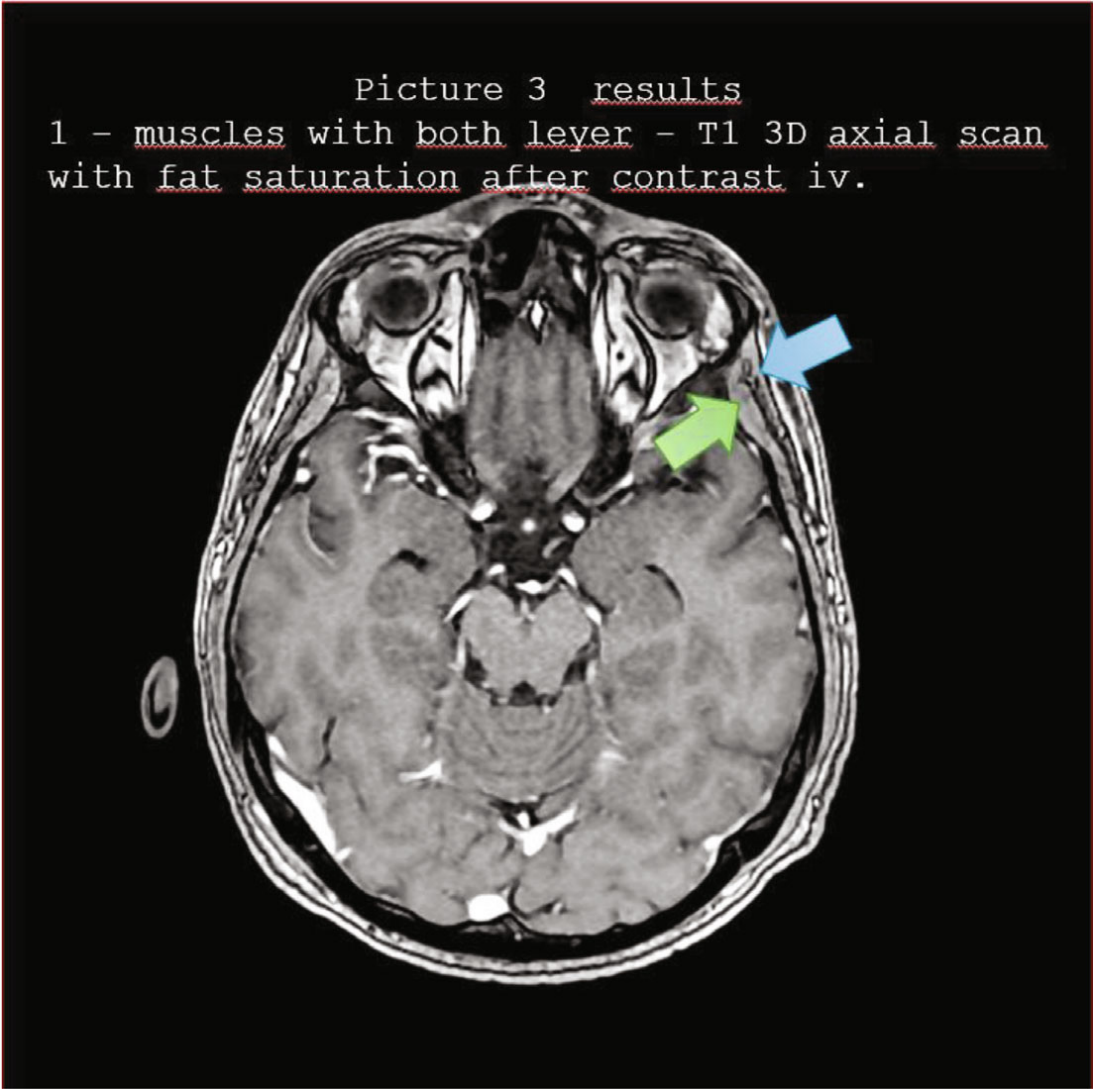
Results 1—muscles with both layers—T1 3D axial scan with fat saturation after contrast IV.

**Figure 4:**
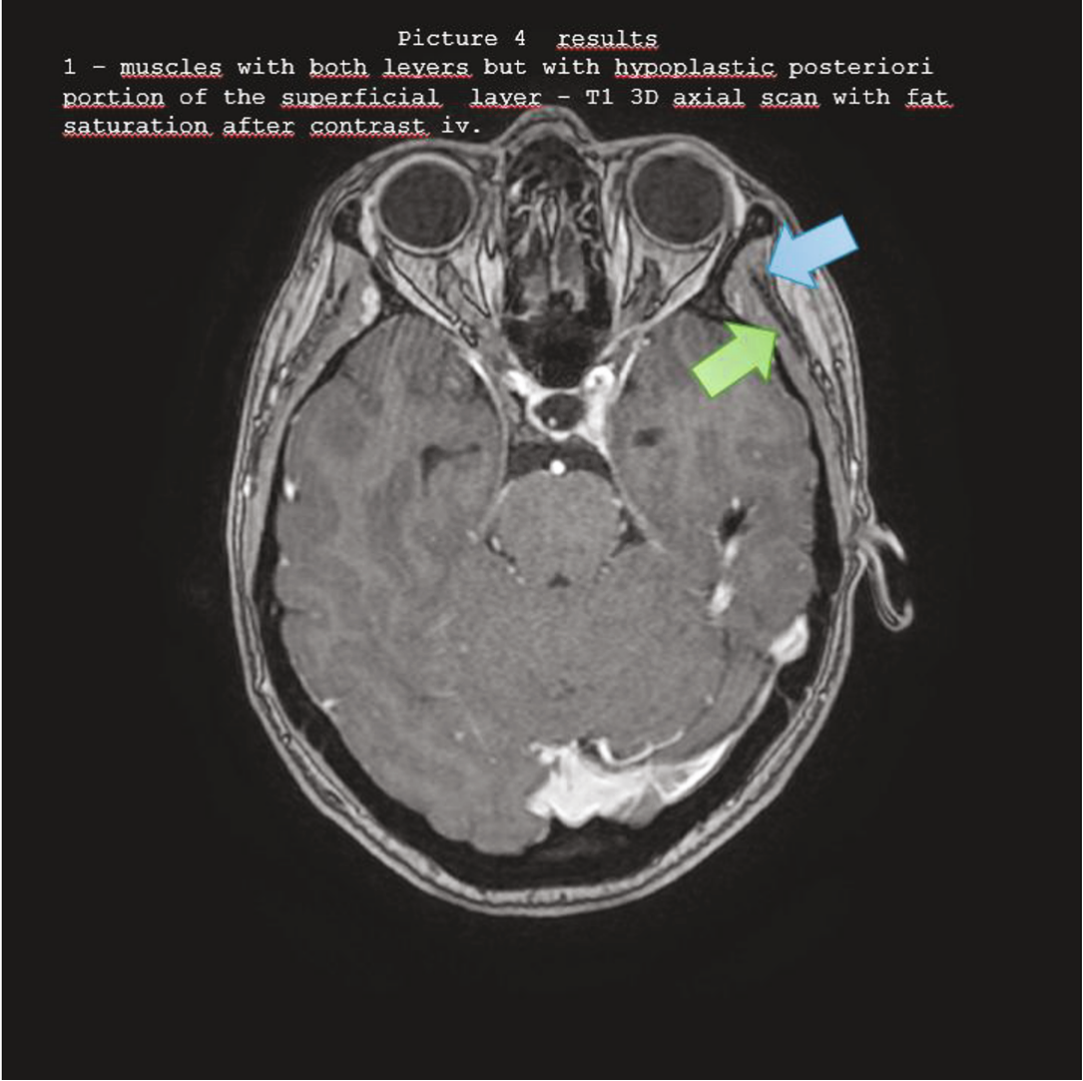
Results 1—muscles with both layers but with hypoplastic posterior portion of the superficial layer—T1 3D axial scan with fat saturation after contrast IV.

**Figure 5:**
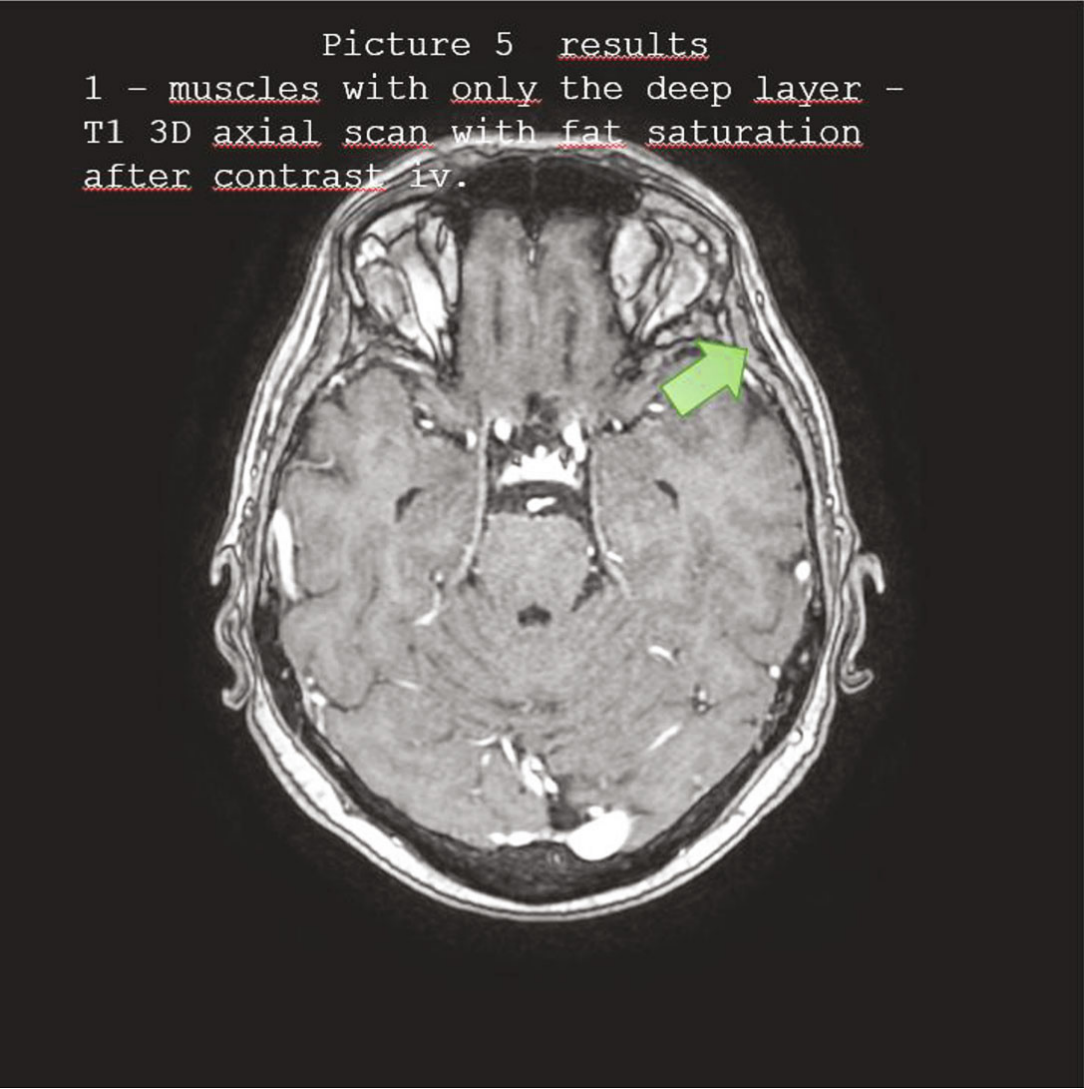
Results 1—muscles with only the deep layer—T1 3D axial scan with fat saturation after contrast IV.

Figures 1, 2, 3, 4, and 5 illustrate representative configurations and measurement planes.

A comparison of morphological data across the three types is presented in Table 1.

The deep layer thickness differed significantly across types (Kruskal–Wallis: *p* = 0.0048 at the MCF level; *p* = 0.0021 for maximum thickness). Descriptively, Type 3 showed the highest median values. Sex‐ and side‐specific morphometric data are presented in Table 2.

In the entire cohort, the length of the coronoid process correlated with the distance from the coronoid apex to the temporalis attachment (Spearman’s rank correlation, *p* = 0.0001). Additional associations were observed with the superficial‐layer thickness and the overall muscle length measured at the MCF plane (*p* = 0.0191 and *p* = 0.0230, respectively), as well as with the deep layer’s maximum length and maximum height (*p* = 0.0150 and *p* = 0.0468, respectively).

## 4. Discussion

In the current study, we assessed the morphological variability of the TM with MRI scans. Muscle with two morphologically similar layers was the most common finding in our sample (72.4%), while the absence of the superficial layer appeared in 5.2%. These results align with previous cadaveric and radiological findings [1, 9].

Although the TM was reported to have few variants in older anatomical compilations, recent studies have revealed a more complex anatomical structure [4, 9, 10]. Geers et al. [9] confirmed the presence of distinct superficial and deep portions of the TM through cadaveric and MRI‐based observations, including in 100 clinical cases. Our findings expand on this by documenting cases of complete absence of the superficial layer, an anomaly not described in their material, possibly due to our larger and more diverse MRI‐based sample.

Other researchers have suggested that the TM can be divided into anterior and posterior components [11], or even into superficial, zygomatic, and deep complex layers [10]. The theory of a transitional muscular bundle connecting the TM with the lateral pterygoid muscle has also gained attention [12]. Hanasono et al. [13] identified distinct superficial and deep tendons of the TM inserting at the coronoid process, supporting the theory of complex tendinous anatomy.

Furthermore, recognizing the presence or absence of distinct TM layers is crucial not only for diagnostic and reconstructive purposes, but also for planning facial reanimation procedures. Split or lengthened temporalis myoplasty techniques rely heavily on the integrity and thickness of specific muscle segments, particularly in the posterior and deep fibers, which serve as reliable donors for tendon transposition [14, 15].

The identification of hypoplasia in the posterior portion of the superficial layer in a substantial subset of participants, especially in males, may have functional consequences. Such anatomical features could contribute to compromised mastication, inefficient jaw closure, or imbalances in force transmission. Absence or hypoplasia of the superficial layer may also affect the vector of pull on the coronoid process and consequently alter mandibular condyle positioning, potentially exacerbating TMD.

In addition to localized pathology, the TM has recently gained attention as a potential marker of systemic conditions such as sarcopenia. Reduced TM thickness measured on CT or MRI has been correlated with poorer oncologic outcomes and shorter progression‐free survival in head and neck cancer patients [1, 16].

These findings emphasize the clinical importance of recognizing TM variants. Imaging, particularly MRI and CT, plays a vital role in identifying such anatomical features. In cases where physical examination does not reveal muscle asymmetry or structural abnormality, radiological assessment can detect subtle changes such as layer‐specific hypoplasia or absence [1, 6, 16].

Asymmetry or volume loss in the superficial temporalis layer can also impact the postoperative contour of the temporal region, which is relevant when planning PEEK or PMMA implants to prevent temporal hollowing after flap harvest [5, 17].

Understanding these structural differences also has implications for reconstructive and functional facial surgery. The variability in muscle architecture may influence decisions regarding flap harvest, tendon transfer, or facial reanimation procedures [2, 8]. Incorporating detailed anatomical and radiological assessment into preoperative planning enhances diagnostic precision and allows for better personalized treatment strategies [7].

It should be noted that differentiating between the superficial and deep layers of the TM in MRI may be confounded by partial volume effects, low contrast resolution in certain sequences, or variations in muscle angulation. Standardized MRI protocols, including specific T1‐weighted oblique reconstructions, may enhance detection of subtle morphological variants.

This study has limitations. Although a larger sample would have been desirable, the number of TMs analyzed (*n* = 116 hemifaces) exceeds that in most MRI‐based anatomical studies. The cohort was relatively homogeneous, comprising individuals residing in central Poland. No formal sample size calculation was undertaken, which may reduce statistical power—particularly for subgroup analyses.

## 5. Conclusion

In conclusion, this study provides valuable insights into the morphological variability of the TM, revealing significant gender‐based and anatomical differences. The presence of both superficial and deep layers was common, with hypoplasia of the posterior portion of the superficial layer observed more frequently in males, highlighting the importance of understanding these variations in clinical practice. The deeper layer’s increased thickness in individuals with hypoplastic superficial portions suggests a compensatory hypertrophy response, which may have implications for understanding functional adaptations in masticatory muscles, particularly in the context of TMDs. The significant correlation between the length of the coronoid process and muscle attachment points underscores the functional importance of anatomical structures in maintaining optimal muscle function. These findings suggest that variations in the TM’s morphology may be critical in diagnosing and managing conditions such as TMJ dysfunction, bruxism, and other masticatory pathologies. Clinicians should consider these variations when planning surgical procedures or devising treatment strategies, as they may influence both the outcomes and the approach to managing masticatory disorders. Further research is warranted to explore the clinical relevance of these findings in greater detail and their implications for personalized patient care.

## Ethics Statement

Ethical approval was obtained from the Bioethics Committee at the Medical University of Lodz (Approval No. RNN/39/25/KE**),** in accordance with the 1964 Helsinki Declaration and its later amendments.

## Consent

The authors have nothing to report.

## Disclosure

All authors reviewed and approved the final version.

## Conflicts of Interest

The authors declare no conflicts of interest.

## Author Contributions

A.O., I.C.L., M.P., and R.F.: data collection and image analysis; G.T.: literature review and anatomical commentary; Ł.O.: study conception, statistical analysis, and manuscript drafting.

## Funding

No funding was received for this manuscript.

## Data Availability

The datasets generated and/or analyzed during the current study are not publicly available due to privacy and ethical restrictions related to medical imaging data but are available from the corresponding author on reasonable request and upon appropriate institutional/ethical approval.

## References

[1] Lee B. , Bae Y. J. , Jeong W.-J. , Kim H. , Choi B. S. , and Kim J. H. , Temporalis Muscle Thickness as an Indicator of Sarcopenia Predicts Progression-Free Survival in Head and Neck Squamous Cell Carcinoma, Scientific Reports. (2021) 11, no. 1, 10.1038/s41598-021-99201-3, 34611230. PMC8492642 34611230

[2] Brennan T. , Tham T. M. , and Costantino P. , The Temporalis Muscle Flap for Palate Reconstruction: Case Series and Review of the Literature, International Archives of Otorhinolaryngology. (2017) 21, no. 3, 259–264, 10.1055/s-0037-1598653, 2-s2.0-85013170729, 28680495. 28680495 PMC5495588

[3] Hassanein A. G. , Continuous Validity of Temporalis Muscle Flap in Reconstruction of Postablative Palatomaxillary Defects, Journal of Craniofacial Surgery. (2017) 28, no. 2, e130–e137, 10.1097/SCS.0000000000003323, 2-s2.0-85007484163, 28033186. 28033186

[4] Bae H. , Choi Y. J. , Lee K. L. , Gil Y. C. , Hu K. S. , and Kim H. J. , The Deep Temporal Arteries: Anatomical Study With Application to Augmentations Procedures of the Temple, Clinical Anatomy. (2023) 36, no. 3, 386–392, 10.1002/ca.23952, 36136301. 36136301

[5] Laloze J. , Brie J. , Chaput B. , and Usseglio J. , Depression After Temporal Muscle Flap: A Systematic Review of the Literature, Journal of Cranio-Maxillo-Facial Surgery. (2019) 47, no. 7, 1104–1109, 10.1016/j.jcms.2019.03.031, 2-s2.0-85065033876, 31056377. 31056377

[6] Ota Y. , Moore A. G. , Spector M. E. , Casper K. , Stucken C. , Malloy K. , Lobo R. , Baba A. , and Srinivasan A. , Prediction of Wound Failure in Patients With Head and Neck Cancer Treated With Free Flap Reconstruction: Utility of CT Perfusion and MR Perfusion in the Early Postoperative Period, American Journal of Neuroradiology. (2022) 43, no. 4, 585–591, 10.3174/ajnr.A7458, 35361578. 35361578 PMC8993192

[7] Wong Z. Y. , de Jongh F. W. , Ingels K. , van Heerbeek N. , and Pouwels S. , Outcomes of Temporalis Muscle-Based Facial Reanimation Surgery: A Systematic Review and Meta-Analysis, JPRAS Open. (2025) 43, 105–121, 10.1016/j.jpra.2024.10.015, 39698478. 39698478 PMC11652750

[8] Abdelrahman E. M. , Zidan A. M. , Sapri A. S. , Shoulah A. A. , Seif O. , Halawa S. M. , Sobhy M. G. , Tantawy R. F. , Abdelraouf O. R. , and Elgazzar S. A. , The Utility and Outcomes of the Temporalis Muscle Flap for Palate Reconstruction After Maxillectomy for Malignancy, Asian Journal of Surgery. (2025) 48, no. 1, 274–280, 10.1016/j.asjsur.2024.08.245. 39271317

[9] Geers C. , Nyssen-Behets C. , Cosnard G. , and Lengelé B. , The Deep Belly of the Temporalis Muscle: An Anatomical, Histological and MRI Study, Surgical and Radiologic Anatomy. (2005) 27, no. 3, 184–191, 15821860. 15821860 10.1007/s00276-004-0306-3

[10] Sedlmayr J. C. , Kirsch C. F. , and Wisco J. J. , The Human Temporalis Muscle: Superficial, Deep, and Zygomatic Parts Comprise One Structural Unit, Clinical Anatomy. (2009) 22, no. 6, 655–664, 10.1002/ca.20837, 2-s2.0-68549117212, 19637294. 19637294

[11] Kemp W. J.3rd, Tubbs R. S. , and Cohen-Gadol A. A. , The Innervation of the Scalp: A Comprehensive Review Including Anatomy, Pathology, and Neurosurgical Correlates, Surgical Neurology International. (2011) 2. 10.4103/2152-7806.90699 PMC3262995 22276233

[12] Sakaguchi-Kuma T. , Hayashi N. , Fujishiro H. , Yamaguchi K. , Shimazaki K. , Ono T. , and Akita K. , An Anatomic Study of the Attachments on the Condylar Process of the Mandible: Muscle Bundles From the Temporalis, Surgical and Radiologic Anatomy. (2016) 38, no. 4, 461–467, 10.1007/s00276-015-1587-4, 2-s2.0-84946925241, 26566895. 26566895

[13] Hanasono M. M. , Utley D. S. , and Goode R. L. , The Temporalis Muscle Flap for Reconstruction After Head and Neck Oncologic Surgery, Laryngoscope. (2001) 111, no. 10, 1719–1725, 11801932. 11801932 10.1097/00005537-200110000-00009

[14] Labbé D. and Huault M. , Lengthening Temporalis Myoplasty and Lip Reanimation, Plastic and Reconstructive Surgery. (2000) 105, no. 4, 1289–97; discussion 1298, 10.1097/00006534-200004000-00005, 2-s2.0-0034021091, 10744217. 10744217

[15] Moubayed S. P. , Labbé D. , and Rahal A. , Lengthening Temporalis Myoplasty for Facial Paralysis Reanimation: An Objective Analysis of Each Surgical Step, JAMA Facial Plastic Surgery. (2015) 17, no. 3, 179–182, 10.1001/jamafacial.2015.46, 2-s2.0-84941890637, 25764525. 25764525

[16] Lee H. J. , Jung S. J. , Kim S. T. , and Kim H. J. , Ultrasonographic Considerations for Safe and Efficient Botulinum Neurotoxin Injection in Masseteric Hypertrophy, Toxins (Basel). (2021) 13, no. 1, 10.3390/toxins13010028. PMC7824038 33406757

[17] Ali S. , Abdel Aziz O. , and Ahmed M. , Patient-Specific PEEK Implants for Immediate Restoration of Temporal Fossa After Maxillary Reconstruction With Temporalis Muscle Flap, Maxillofacial Plastic and Reconstructive Surgery. (2022) 44, no. 1, 10.1186/s40902-022-00348-4, 35524015. PMC9076787 35524015

